# Hyperspectral Imaging and the Retina: Worth the Wave?

**DOI:** 10.1167/tvst.9.9.9

**Published:** 2020-08-05

**Authors:** Sophie Lemmens, Jan Van Eijgen, Karel Van Keer, Julie Jacob, Sinéad Moylett, Lies De Groef, Toon Vancraenendonck, Patrick De Boever, Ingeborg Stalmans

**Affiliations:** 1 University Hospitals UZ Leuven, Department of Ophthalmology, Leuven, Belgium; 2 KU Leuven, Biomedical Sciences Group, Department of Neurosciences, Research Group Ophthalmology, Leuven, Belgium; 3 VITO (Flemish Institute for Technological Research), Health Unit, Boeretang, Belgium; 4 Department of Psychiatry, University of Cambridge School of Clinical Medicine, Cambridge Biomedical Campus, Cambridge, UK; 5 Neural Circuit Development and Regeneration Research Group, Department of Biology, KU Leuven, Leuven, Belgium; 6 Hasselt University, Centre of Environmental Sciences, Agoralaan, Belgium

**Keywords:** hyperspectral imaging, retina, eye, oximetry, Alzheimer

## Abstract

**Purpose:**

Hyperspectral imaging is gaining attention in the biomedical field because it generates additional spectral information to study physiological and clinical processes. Several technologies have been described; however an independent, systematic literature overview is lacking, especially in the field of ophthalmology. This investigation is the first to systematically overview scientific literature specifically regarding retinal hyperspectral imaging.

**Methods:**

A systematic literature review was conducted, in accordance with PRISMA Statement 2009 criteria, in four bibliographic databases: Medline, Embase, Cochrane Database of Systematic Reviews, and Web of Science.

**Results:**

Fifty-six articles were found that meet the review criteria. A range of techniques was reported: Fourier analysis, liquid crystal tunable filters, tunable laser sources, dual-slit monochromators, dispersive prisms and gratings, computed tomography, fiber optics, and Fabry-Perrot cavity filter covered complementary metal oxide semiconductor. We present a narrative synthesis and summary tables of findings of the included articles, because methodologic heterogeneity and diverse research topics prevented a meta-analysis being conducted.

**Conclusions:**

Application in ophthalmology is still in its infancy. Most previous experiments have been performed in the field of retinal oximetry, providing valuable information in the diagnosis and monitoring of various ocular diseases. To date, none of these applications have graduated to clinical practice owing to the lack of sufficiently large validation studies.

**Translational Relevance:**

Given the promising results that smaller studies show for hyperspectral imaging (e.g., in Alzheimer's disease), advanced research in larger validation studies is warranted to determine its true clinical potential.

## Introduction

Changes in retinal reflectance and absorbance of light that occur during the development of various eye diseases play a pivotal role in ophthalmologic diagnostics. Spectral information obtained from digital retinal images is limited; only monochromatic or trichromatic (red, green, blue) light reflected by retinal structures is registered.^1^ Hyperspectral imaging (HSI) technology has the potential to overcome this shortcoming by producing an image that captures information from multiple wavelengths, generating a four-dimensional hyperspectral cube: two dimensions for orthogonal spatial data, a third for wavelength bands, and finally one for their corresponding absorbance/reflectance intensities at each wavelength. Hyperspectral imagery collects data from tens to hundreds of narrow wavelength bands, whereas multispectral imagery refers to data from 3 to 10 wider bands. After illumination of the retina, the reflected light is captured via spectrometers, allowing spectral analysis to be performed on the hyperspectral cube.^2^ HSI allows considerably more spectral information to be collected compared with conventional retinal imaging; however, advances in this technology within biomedicine, and specifically in ophthalmology, are only emerging.

Examples of HSI usability within (bio)medical disciplines range from perioperative support with guidance of the surgeon to delineate the right resection margins of lentigo maligna or cerebral neoplasms to assessing diabetic foot ulcer development risk.^2^^–^^6^ Other proof-of-concepts measured the oxygen saturation (OS) of various organs^7^; assessed the presence of molecules such as cholesterol, melanin, and hemoglobin^8^^,^^9^; enhanced the surgeon's vision in oncologic surgery and laparoscopy^10^^–^^13^; predicted hemorrhagic shock and appraising hemodynamics^14^^–^^16^; classified corneal injury^17^; augmented contrast for histologic examinations^18^^,^^19^; and detected neoplasms of the skin, mouth, colon, brain, and others.^3^^,^^5^^,^^20^^–^^22^ In ophthalmology, HSI can be used to assess the state and distribution of chromophores, such as cytochrome C, and assess the metabolic status of hemoglobin in the context of retinal blood vessel oxygenation. Such information is valuable to diagnose and monitor various ocular diseases including age-related macular degeneration (ARMD), glaucoma, and diabetic retinopathy.^23^^–^^33^

For further adoption of HSI technology in ophthalmologic research, it is important to explain the underlying imaging principles and describe the current relevant techniques, allowing for the identification of gaps and directions for future research and practice. Retinal HSI for ophthalmology has been documented before, but only included in reviews that analyzed the entire medical field or in manuscripts that date from several years ago.^2^^,^^26^^,^^31^^,^^34^^–^^36^ This article provides an up-to-date systematic review of HSI of the retina and explores how this technique can contribute to the diagnosis and monitoring of a range of ophthalmologic and systemic conditions.

## Methods

### Literature Search

A literature search was conducted independently by two reviewers (SL and JVE) in four databases: Medline (through PubMed), Embase, the Cochrane Database of Systematic Reviews, and Web of Science. Boolean operators with the widest coverage were selected (Appendix A). The Boolean operator for PubMed is presented here as an example:

((“eye”[MeSH] OR eye*[tiab] OR “ophthalmology”[MeSH] OR ophthalmology*[tiab] OR “retinal vessels”[MeSH] OR retina*[tiab] OR fundus*[tiab] OR macula*[tiab] OR fovea*[tiab])) AND hyperspectral*[tiab].

The last search was conducted on January 1, 2020, producing a flow chart (Supplementary Fig. S1) in line with the PRISMA statement.^37^ Articles were screened initially for relevance by their title and abstract, and secondly by their full text. Inconsistencies were solved by consensus between the two reviewers (SL and JVE).

### Selection and Exclusion Criteria

All study designs were accepted after applying the exclusion criteria. The following exclusion criteria were used: (i) all tissues except retina; (ii) imaging techniques that are not hyperspectral or that are a hyperspectral extension of an essentially different method; (iii) studies on the chemical properties of photoreceptors or photochemistry; (iv) meeting abstracts; (v) reviews without experimental additions; and (vi) studies written in languages other than English. The latest article was included when redundancy occurred in literature.

### Quality Assessment

From all studies, the following details were extracted (when mentioned): (1) author, (2) journal, (3) year of publication, (4) study design, (5) number of participants, (6) average age of participants, (7) main pathology, (8) hardware, (9) outcome, (10) acquisition time, (11) spectral and spatial resolution, and (12) field of view. When possible, the Newcastle-Ottawa Quality Assessment Scale (for case-control or cohort studies) or the Quality Assessment of Diagnostic Accuracy Studies-2 tools were used.^38^ Selection, comparability, and exposure were evaluated in the Newcastle-Ottawa Quality Assessment Scale. Risk of bias and applicability were evaluated for patient selection, index test, reference standard, and flow and timing in Quality Assessment of Diagnostic Accuracy Studies-2.^39^ JVE and SL extracted and calculated these data and made the quality assessments independently. Publication bias was unraveled in the best possible way. Discrepancies were resolved by consensus.

## Results

### Identification and Quality Assessments of Publications

The majority of the records were identified through Web of Science (*n* = 382), followed by Embase (*n* = 194), Medline (*n* = 127), and lastly the Cochrane Database of Systematic Reviews (*n* = 3) (flow chart in Supplementary Fig. S1). Four articles identified through a review were added.^2^^,^^29^^,^^40^^,^^41^ A total of 361 articles were screened using the described selection and exclusion criteria. Finally, 56 eligible articles were identified for inclusion in the systematic review (Supplementary Table S1). Information about the quality assessment (12 papers) is annexed (Supplementary Tables S2 and S3).

### HSI Techniques

Spectral imaging is defined as the technology that combines conventional imaging and spectroscopy methods to obtain both spatial and spectral information of an object.^42^ Notably, not all techniques that are claimed to be “hyperspectral” achieve dozens to hundreds of spectral bands (Table 1), the requisite amount according to the definition of HSI by Gao et al.^35^ A typical HSI set-up consists of a light source that emits light directed toward the retina (incident light, illumination path) that is then partly reflected and transmitted by the retina (reflected/transmitted light, imaging path). Depending on the set-up mode, the reflected or transmitted light can be captured by a detector, most commonly a charge coupled device (CCD).

**Table 1.** Technical Specifications per Paper, Ordered per HSI Style

	Year	Study Type	Pathology	Hardware	Snapshot	No. of Participants	Wavelength Range (Resolution), Pixels (Resolution)
Truitt et al.^48^	2000	Exp human in vivo	NA	FTVHSI (CCD + rotating mirror)	No (8.3 fps, 1 D)		450–800 nm 54 bands (4–11 nm), (56 × 79 µm retina per pixel)
Zamora et al.^68^	2004	Case control	DM CSME	HSFI (CCD)	No (1 D)	2	400–800 nm 110 bands (4–5 nm)
Davis et al.^69^	2007	Case control	Different macular diseases	HSRID (CCD)	No (1 D)	15 + 20 co	500–700 nm 100 bands (20 nm), 1 line of 50 × 200 µm
Schweizer et al.^29^	2012	Exp in vitro	AMD	Fourier transform interferometer (CCD + microscope)	No (50 fps)		1. 400–600 per 10 nm (7 nm)
Harvey et al.^49^	2002	Exp human in vivo	DR + glaucoma	Band-pass interference filters + CCD	No	3	400–1100 nm, 1024 × 1344 pixels (illumination)
Alabboud et al.^51^	2007	Exp human in vivo	Healthy oximetry	(1) LCTF + CCD (2) IRIS	1. No 2. Yes^a^		(1) 400–700 nm per 7–9 nm (2) 560–600 nm and 577–600 nm 8 bands
Mordant et al.^52^	2011	Exp in vitro	Oximetry	LCTF + CCD	No <5 min		500–650 nm per 2 nm
Mordant et al.^26^	2011	Exp human in vivo	Healthy Oximetry	LCTF + CCD	No	14 (+1 RVO)	500–650 nm per 2 nm (420–720 nm per 10 nm also possible)
Mordant et al.^53^	2014	Case control	POAG	LCTF + CCD	No	11 POAG + 14 co	556–650 nm per 2 nm
Nourrit et al.^28^	2010	Exp human in vivo	DM + glaucoma oximetry	LCTF + CCD	No <1.6 s	28 (POAG + DR + NL)	495–720 nm at 8 predetermined wavelengths, 336 × 256 pixels
Hirohara et al.^54^	2007	Exp human in vivo	Healthy oximetry	LCTF + CCD	No 7 s (4.9 fps)	16	500–720 nm per 10 nm (20 nm), 320 × 256 pixels
Tam et al^.^^64^	2011	Exp mice in vivo	Glaucoma (lymphatics)	LCTF + CCD (MaestroTM) + microscope	No (1.1 fps)		500–800 nm per 10 nm
Smith et al.^47^	2014	Exp human ex vivo	Healthy RP	LCTF + CCD (Nuance Fx Multispectral camera) + microscope	No	20	420–720 nm per 10 nm
Ben Ami et al.^46^	2016	Exp human ex vivo	Healthy RP	LCTF + CCD (Nuance Fx Multispectral camera) + microscope	No	20	420–720 nm per 10 nm (excited at 2 bands, 436–460 and 480–510 nm)
Tong et al.^33^	2016	Exp human ex vivo	AMD RP	LCTF + CCD (Nuance Fx Multispectral camera) + microscope	No	5	420–720 nm per 10 nm (excited at 2 bands, 436–460 and 480–510 nm)
Dey et al.^65^	2019	Exp human ex vivo	AMD RP	LCTF + CCD (Nuance Fx Multispectral camera) + microscope	No	15	420–720 nm per 10 nm (excited at 436, 480, 500 and 560 nm)
Francis et al.^66^	2011	Exp human in vivo	AMD + hyperbilirubinemia	TLS + CCD	No		(1.5 nm)
Patel et al.^57^	2013	Exp human in vivo	Healthy oximetry	TLS + CCD	No 10 s (12.5 fps)	6	500–600 nm 5 bands, 1392 × 1040 pixels
Shahidi et al.^60^	2013	Exp human in vivo	Healthy oximetry	TLS + CCD	No 3 s (12.5 fps)	9	500–650 nm 5 bands, 1392 × 1040 pixels
Shahidi et al.^30^	2017	Case control	POAG	TLS + CCD	No 3 s (12.5 fps)	22 POAG + 17 co	548–610 nm 5 bands
Tayyari et al.^32^	2015	Case control	DM oximetry	TLS + CCD	No (12.5 fps)	13 DM + 15 co	548, 569, 586, 600, 605, et al. and 610 nm
Tayyari et al.^62^	2019	Case control	DR oximetry	TLS + CCD	No (12.5 fps)	14 DR + 17 co	548, 569, 586, 600, 605, et al. and 610 nm
Rose et al.^58^	2016	Exp human in vivo	Healthy oximetry	TLS + CCD (MHRC)	No (27 fps)	11	500–650 nm per 5 nm, 1.3 megapixel
Desjardins et al.^63^	2016	Exp human in vivo	Glaucoma + healthy ox.	TLS + CCD (MHRC)	No <3 s (27 fps)	2 glaucoma + 11 co	500–600 nm per 2 and 5 nm, 1.3 megapixel
Rose et al.^59^	2018	Case control	Radiation retinopathy	TLS + CCD (MHRC)	No	8	520–620 nm per 5 nm
Hadoux et al.^56^	2019	Case Control	Alzheimer	TLS + CCD (MHRC)	No 1 s (100 fps) frames)	15 + 20 co	450–900 nm per 5 nm
Sharafi et al.^61^	2019	Case control	Alzheimer	TLS + CCD (MHRC)	No 1 s (91 frames)	16 + 30 co	450–900 nm per 5 nm
More et al.^67^	2016	Exp mice in vivo	Alzheimer	Monochromator + CCD	No 20 s	8 per group	408–705 nm 16 bands (15 nm), 1392 × 1024 pixels
Khoobehi et al.^50^	2012	Exp human in vivo	Healthy oximetry	Lens array + 7 band-pass filters + CCD	Yes		
Khoobehi et al.^70^	2004	Exp monkey in vivo	Healthy oximetry	Spectrograph (PGP architecture + CCD) + linear actuator	No 10–30 s	2	410–950 nm 256 bands (2.5 nm), 512 pixels
Beach et al.^71^	2007	Exp monkey in vivo	Oximetry with varying IOP	Spectrograph (PGP architecture + CCD) + linear actuator	No 10–30 s	5	384 × 384 pixels
Beach et al.^72^	2009	Exp monkey in vivo	Oximetry with varying IOP	Spectrograph (PGP architecture + CCD) + linear actuator	No 10–30 s		385 × 384 pixels
Khoobehi et al.^73^	2009	Exp monkey in vivo	Oximetry with varying IOP	Spectrograph (PGP architecture + CCD) + linear actuator	No 10–30 s		386 × 384 pixels
Khoobehi et al.^74^	2011	Exp monkey in vivo	Oximetry under NCX 434	Spectrograph (PGP architecture + CCD) + linear actuator	No		
Liu et al.^75^	2012	RCT mice in vivo	Cerebral malaria	?	No		
Li et al.^43^	2007	RCT rats ex vivo	DR	Spectrometer (PGP) + CCD + microscope + linear actuator	No	12 DM + 10 co	404–865 nm 240 bands (2 nm), 460 × 300 pixels
Li et al.^44^	2008	RCT rats ex vivo	DR	Spectrometer (PGP) + CCD + microscope + linear actuator	No		400–800 nm 240 bands (2 nm), 640 × 300 pixels (1.125 µm)
Li et al.^45^	2010	RCT rats ex vivo	DR + EPO	Spectrometer (PGP) + CCD + microscope + linear actuator	No	40	400–780 nm, (0.3–0.6 µm)
Gao et al.^76^	2012	Exp human in vivo	Healthy oximetry + MP	IMS (mirror array + prism array + CCD)	Yes (5.2 fps)	1	470–650 nm 48 bands (4 nm), 350 × 350 pixels
Dwight et al.^23^	2016	Case series	AMD, ReP + chron. IC	IMS (mirror array + prism array + CCD)	Yes (5 fps)	4	470–670 nm 40 bands (4 nm), 350 × 350 pixels
Dwight et al.^77^	2019	Exp human in vivo	Oximetry	IMS (mirror array + prism array + CCD)	Yes (5 fps)	15	470–670 nm 43 bands (4.7 nm), 350 × 350 pixels
Yamauchi et al.^78^	2012	Case control	AMD	HSI NIR (mirror + spectrograph + CCD)	No 5 s (60 fps)	62 AMD + 12 co	412–1033 nm 640 bands (0.97 nm), 480 × 321 pixels (33 µm × 16 µm)
Kameyama et al.^79^	2015	Diagnostic Accuracy	Choroidal Melanoma	HSI NIR (mirror + spectrograph + CCD)	No 5 s	5 CM + 12 co	720–950 nm (0.97 nm), (33 µm vertically and 16 µm horizontally)
More et al.^27^	2015	Exp mice ex vivo	Alzheimer	PGP + CCD + microscope + linear actuator	No		400–1000 nm 467 bands (2.5 nm), 322× 322 nm retina per pixel
Browne et al.^80^	2017	Exp In vitro	hESC + iPSC cultures	Hspec (excitation at 740 nm) + Microscope	No 15–20 s		420–690 nm 64 bands (excited at 740 nm)
More et al.^81^	2019	Case control	Alzheimer	Beam splitter + CCD and spectrograph	Yes (1 line)	19 + 16 co	400–1000 nm per 2.5 nm
Johnson et al.^36^	2007	In silico + 1 case/co	Healthy oximetry	CTIS + CCD	Yes 3 ms		450–700 nm 50 bands
Fawzi et al.^24^	2011	Exp human in vivo	Healthy MP	CTIS + CCD	Yes 20 ms	6	420–720 nm 76 bands (4 nm), 186 × 186 pixels (22 µm)
Kashani et al.^25^	2011	Exp rats in vivo	Healthy oximetry	CTIS + CCD	Yes 3 ms		450–700 nm 76 bands, (vessels to 50 µm)
Jaime et al.^82^	2012	Exp rats in vivo	RVO oximetry	CTIS + CCD	Yes	30	
Kashani et al.^83^	2014	Case control	DM oximetry	CTIS + CCD	Yes	12 DM + 45 co	450–700 nm 76 bands (4 nm)
Khoobehi et al. ^84^	2012	Exp human in vivo	Healthy oximetry	648 fibers + 4-split spectrometer + CCD	Yes		(1 nm), 458 (648) pixels (10 µm [20 µm])
Khoobehi et al.^85^	2014	Letter to the Editor	N/A	480 fibers + 4-split spectrometer + CCD	Yes		(1 nm), 458 (648) pixels (10 µm [20 µm])
Li et al.^86^	2017	Exp in vitro + rat in vivo	Healthy oximetry	SRDA (Fabry–Perot cavity filter + CMOS)	Yes 50 ms		460–630 nm 16 bands (11–19 nm), 256 × 512 pixels (22 µm)
Kaluzny et al.^87^	2017	Exp human in vivo	Healthy oximetry + MP OD	SRDA (Fabry–Perot cavity filter + CMOS)	Yes	12	460–630 nm
Wang et al. et al.^88^	2019	Exp human in vivo	AMD	SRDA (Fabry–Perot cavity filter + CMOS)	Yes	22 AMD + 6 co	460–630 nm 16 bands (11-19 nm), 256 × 512 pixels (22 µm)

CCD, charge-coupled device; chron. IC, chronic iridocyclitis; CM, choroidal melanoma; CMOS, complementary metal oxide semiconductor; co, controls; CSME, clinically significant macular edema; DM, diabetes mellitus; DR, diabetic retinopathy; EPO, erythropoietin; Exp, experimental setting; fps, frames per second; FTVHSI, Fourier Transform hyperspectral imager (Sagnac interferometer + CCD + rotating mirror); hESC, human embryonic stem cell; HFSI, hyperspectral fundus imager (= Fourier Transform Imaging Spectrometer (FTIS) + xenon flashlamp); HSI NIR, hyperspectral imager near-infrared (motor-driven scanning mirror + imaging spectrograph with volume-type holographic transmission grating + CCD); HSRID, hyperspectral retinal imaging device (= Fourier transform spectrometer (FTS) + Sagnac MP: macular pigment study; IMS, imaging spectrograph (interferometer + Fourier lens + cylindrical lens + CCD); IOP, intraocular pressure; iPSC, induced pluripotent stem cell; N/A, not applicable; OD, optical density study; ox., oximetry; POAG, primary open-angle glaucoma; ReP, retinitis pigmentosa; RP, retinal pigment study; RVO, retinal vein occlusion; TLS, tunable wavelength laser source.

^a^Eight bands.

Note: Maestro, Nuance Fx, MHRC, and Hspec are commercially available hyperspectral imagers.

**Figure 1.**
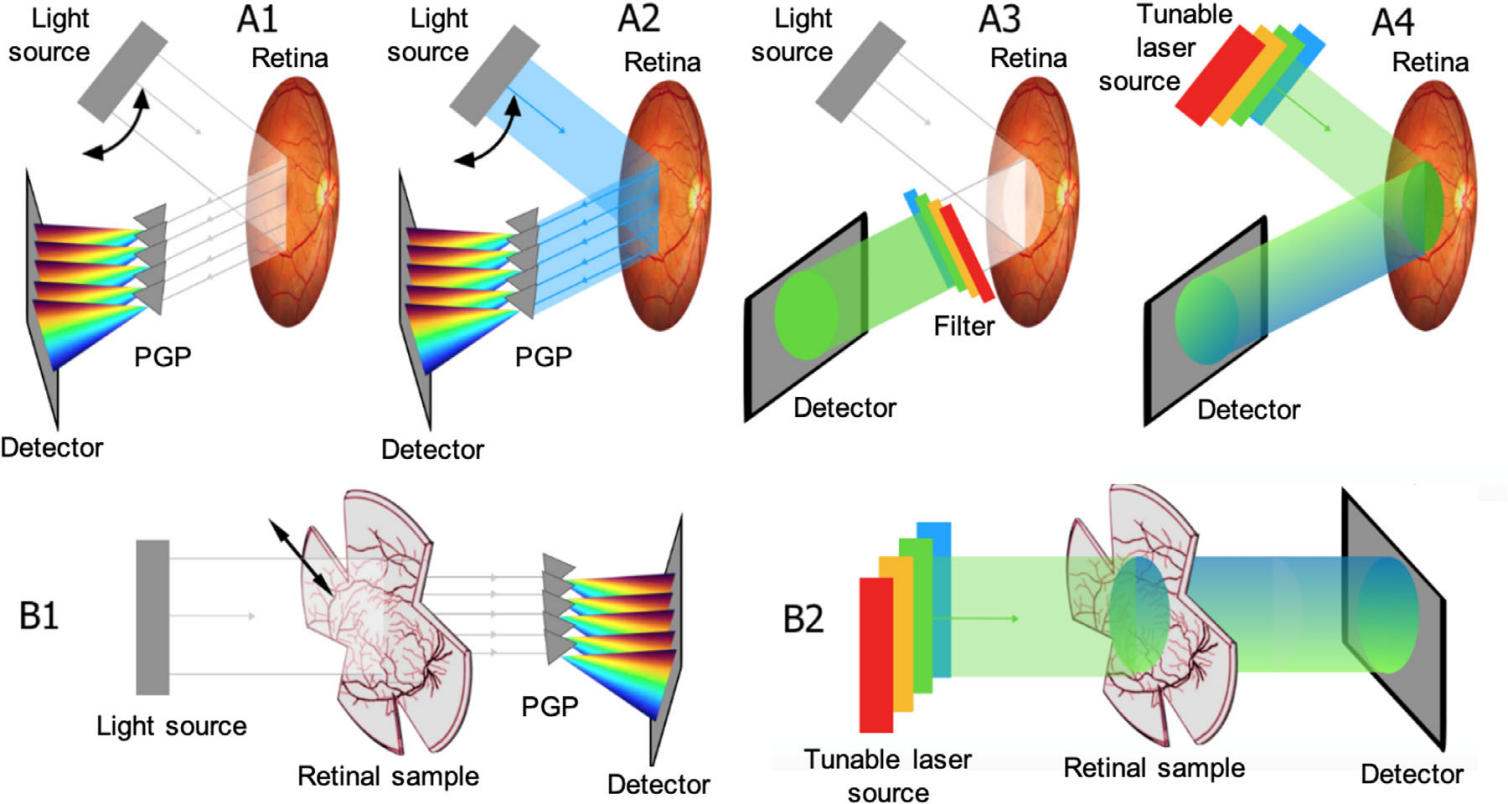
Illustration of different hyperspectral modes. (A) In vivo set-ups for retinal HSI in reflectance mode: (A1) HSI with a PGP (*grey triangles*); (A2) identical to the set-up in A1 but in autofluorescence mode; The double black arrow indicates the turning movement of the illumination source. (A3) HSI with LCTF in imaging path and (A4) HSI method based on LCTF in illumination path or based on TLS. (B) Ex vivo set-ups for retinal HSI in transmission mode. Set-up (B1) is based on a PGP array analogous to A1. (B2) shares an identical illumination as in A4. The color gradient in A4 and B2 refer to Stokes shift.

The choice between transmittance or reflectance mode depends on the accessibility of the imaged tissue (Fig. 1). All cited ex vivo microscopy techniques are designed in transmittance mode (Fig. 1B), that is, detectors capture light transmitted through the retina,^27^^,^^29^^,^^43^^–^^45^ except for those who used an autofluorescent reflectance mode to study the retinal pigment epithelium (RPE) and inherent fluorophores (as discussed elsewhere in this article).^33^^,^^46^^,^^47^ For in vivo imaging, reflectance mode (Fig. 1A) is used, meaning that detectors capture reflected light returned back from the retinal surface.

To achieve multiple wavelength acquisition, several set-ups were developed (Fig. 1). Bandpass filters that pass wavelengths within a certain range and attenuate wavelengths outside that range (e.g., liquid crystal filters) can be placed in the incident beams (Fig. 1A4) and/or reflected beams (Fig. 1A3). Reflected beams could alternatively be dispersed through a prism-grating-prism array (PGP), giving rise to the rainbow-like separation of wavelengths, that is, colors (Figs. 1A1 and A2).

Furthermore, diverging approaches for illumination of the retinal surface are defined, that is, incident light covering the visible spectrum—even to near infrared (Figs. 1A1, A3, B1), part of the spectrum (Figs. 1A4, B2), or specific wavelengths (Fig. 1A2). The latter being illustrated by autofluorescence mode (section A2 of Fig. 1), where the retina is illuminated in a specific blue band, commonly (although not depicted) with optional blocking of shorter wavelengths in the imaging path. Routinely used fundus autofluorescence is based on the intrinsic fluorescence at 488 nm of the retinal fluorophores such as lipofuscin. Autofluorescence mode can be used in ex vivo (e.g., Fig. 1B1) as in vivo set-ups (Fig. 1A3).

**Figure 2.**
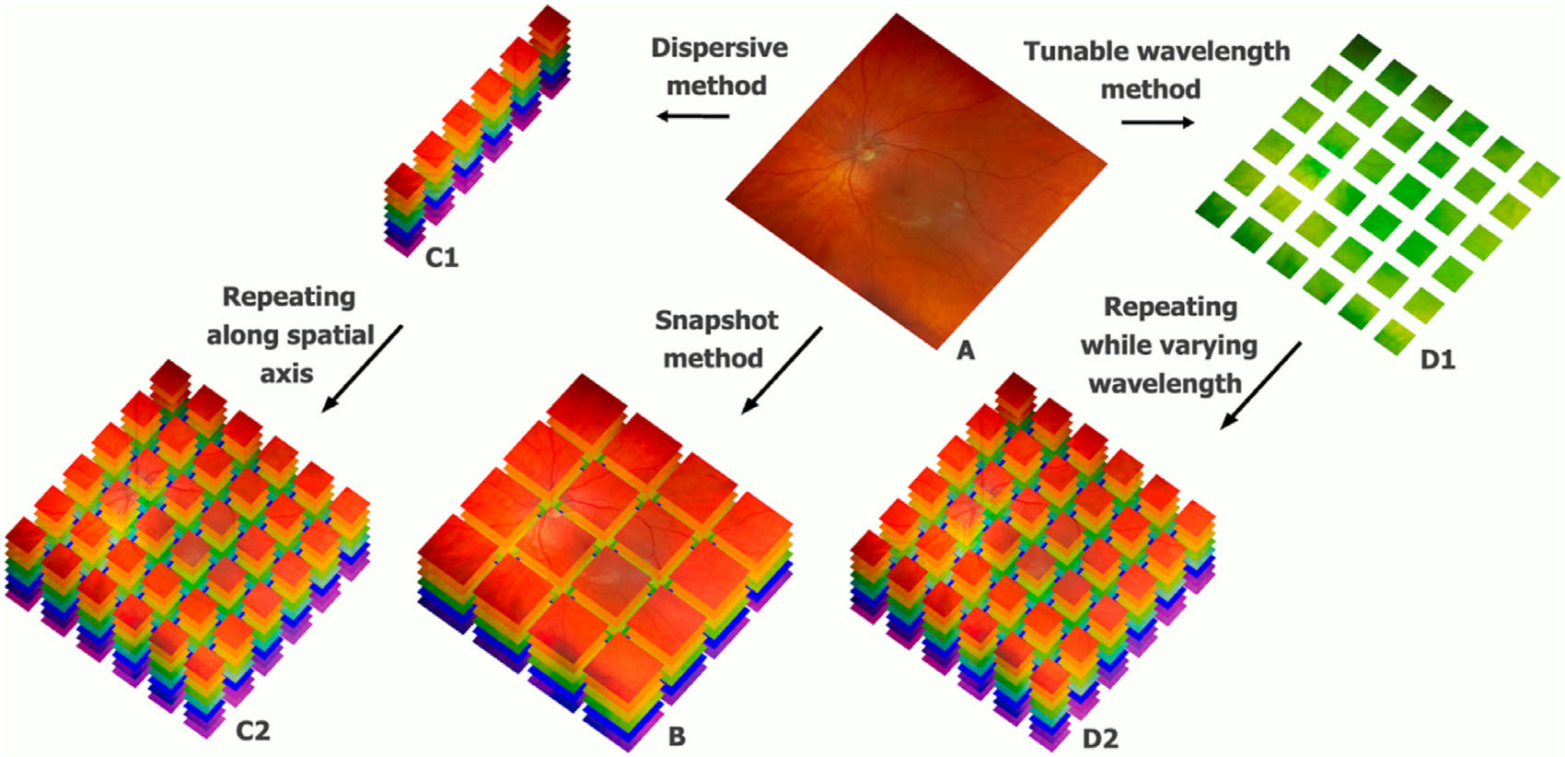
Illustration of the three principal hyperspectral acquisition concepts. Pixels of the fundus (A) are imaged at multiple wavelengths, creating hyperspectral cubes (B, C2, D2). Whereas snapshot techniques (B) produce their hyperspectral cube at once, tunable wavelength methods and dispersive methods capture the retina wavelength by wavelength (D1), and PGP methods capture all wavelengths of a single line and repeat the process along the width of the retina (C1), resulting in higher spatial and spectral resolution but also longer acquisition time.

All of these set-ups have in common that they generate a hyperspectral cube (Figs. 2C2, B, D2), a matrix that contains spectral information, that is, transmittance or reflectance as a function of wavelength, for each spatial dimension (*x*, *y*) of the imaged object (the retina). Figure 2 presents the principal cube acquisition concepts. Whether the whole retina is scanned for all wavelengths line after line (Figs. 2C2 via C1) or by capturing the entire retina but wavelength after wavelength (Figs. 2D2 via D1), the resulting cubes are very similar.

Of note, the spectral resolving detector array (SRDA) set-up (not included in Fig. 1, but analogous to Fig. 1A3) depicts a snapshot technique, in which the entire cube is instantly captured, although with lesser spatial and spectral resolution (Fig. 2B). The difference is that each pixel is covered by its own band pass filter instead of the tunable filters in front of the light detector.

Following this conceptual introduction, a historical overview and detailed analysis of the different hyperspectral retinal imaging models is given below. The defining features of each of the set-ups are textually presented (specific technical details can be found in Table 1), followed by common HSI analysis techniques and reported clinical applications.

#### Fourier Analysis (Though Not Dispersive)

HSI featured for the first time in retinal research in 2000, when Truitt et al. integrated a fundus camera, a CCD, and a Sagnac interferometer. A two-dimensional interferogram was captured and converted to a spectral signature by reverse Fourier analysis (Figs. 2, C1 and C2). A rotating mirror advanced the line of the incident light on the retina, enabling scanning of the whole retina image after image.^48^

#### Tunable Wavelength Methods

Tunable wavelength methods (Figs. 2, D1 and D2) were second to be developed in 2002 and allowed filtering of the incident light by band-pass interference filters (Fig. 1A3).^49^ By juxtapositioning an image multiplicating lens array, Khoobehi et al.^50^ simultaneously captured seven wavelengths, each filtered through a different band pass filter. Later, liquid crystal tunable filters (LCTF), whether in the illumination path (Fig. 1A4)^26^^,^^28^^,^^51^^–^^53^ or in the imaging path (Fig. 1A3),^54^ and tunable laser sources (TLS; Fig. 1A3)^30^^,^^32^^,^^55^^–^^63^ became more common and enabled narrow-band imaging. The latter is also being used in transluminance and autofluorescence mode on retinal flatmounts.^33^^,^^46^ LCTF and TLS are among the most used in the history of HSI, with commercially available, ready-to-use imagers on the market.^33^^,^^46^^,^^47^^,^^56^^,^^58^^,^^59^^,^^61^^,^^63^^–^^65^

Less common ways to tune the wavelength output of a device is the acousto-optic tunable filter, where the diffraction of crystals is altered by acoustic waves (Fig. 1A3),^66^ and a dual-slit scanning monochromator (Fig. 1A3).^67^

#### Dispersive Prisms and Gratings

Next to the human *in vivo* use of PGP arrays, introduced by Khoobehi et al.,^70^^–^^74^ dispersive prisms and gratings are commonly adapted for microscopes (Fig. 1, A1; Figs. 2, C1 and C2), with the microscopic pushbroom HSI system as an example (Fig. 1, B1).^43^^–^^45^ As can be remarked in Table 1, most set-ups that extend their spectrum toward the visible and near-infrared spectrum (ca. 400–1100 nm) belong to this category.^27^^,^^78^^,^^79^ Gao et al.^76^ used a tilted angle image mapper to create a snapshot technique based on PGP.

#### Computed Tomographic Imaging Spectrometer (CTIS) (Fig. 2B)

Yet another type of acquisition is constituted by CTIS: the whole two-dimensional retinal image is simultaneously dispersed in various projections by a diffractive grating, thus representing each pixel several times onto the CCD detector, with each presentation showing a different mix of spatial and spectral data. Software is then used to calculate the exact spectrum per pixel. This process results in a snapshot acquisition of the retinal hypercube.^24^^,^^25^^,^^36^^,^^82^^,^^83^

#### Fiber Optic Cables

In 2012 and 2014, a snapshot HSI technique by rearranging a fiber bundle from a fundus camera to a multislit, grating-type spectrograph was reported.^84^^,^^85^ However, as Fourier techniques and CTIS, fiber optics were not featured in literature during the last 5 years. This is possibly due to the low resolution (in the order of 25 × 25 pixels) that prevents differentiation between choroid and retina, making this technique insufficiently performant for retinal imaging.^50^^,^^85^

#### SRDA (Fig. 2B)

Last, the snapshot technique SRDA (see the Introduction) is the only published technique that makes use of a complementary metal oxide semiconductor (CMOS) detector array instead of a more expensive CCD array.^86^^–^^88^

### Hyperspectral Data Processing

Hyperspectral raw data cubes are typically preprocessed before data analysis. Several studies normalize raw data to obtain relative reflectances using a white and/or dark reference.^24^^,^^26^^,^^36^^,^^57^^,^^58^^,^^76^^,^^85^ Image registration techniques can be subsequently applied to correct for motion artefacts, typically when the acquisition time is longer.^26^^,^^28^^,^^54^^,^^57^^,^^58^ Binning is used to trade off spatial or spectral resolution for light sensitivity.^28^^,^^44^^,^^70^ In the case of spatial binning, for example, charges incident to a 2 × 2 block of pixels can be combined into one ‘binned’ pixel to improve signal-to-noise ratio, at the cost of halving spatial resolution. A comprehensive discussion of the HSI data analysis techniques goes beyond the scope of this review. However, a commonly used data analysis technique, namely, spectral unmixing, is explained. In HSI, each individual pixel consists of a mixture of reflectance spectra of different constituents, also called endmembers. Unmixing aims to identify the endmember spectra, which are typically unknown a priori, and to quantify the abundance of each endmember in each pixel. In most instances, unmixing applies variants of non-negative matrix factorization. Non-negative matrix factorization decomposes the hyperspectral cube into a product of a matrix describing endmembers and a matrix describing the abundance of each endmember to each pixel. Other investigators have applied non-negative tensor factorization, an extension of non-negative matrix factorization that allows a simultaneous decomposition of multiple hyperspectral cubes in the context of autofluorescence HSI with multiple excitation bands.^33^^,^^46^^,^^47^^,^^65^

The upcoming research in neurodegenerative disease (see the Alzheimer's Disease section for an elaboration on the clinical findings) opened up specific retinal HSI analysis for demonstrating and quantifying retinal amyloid burden. More et al.^27^ provide evidence for the presence of beta-amyloid in retina of mice with Alzheimer's disease (AD) by using the angle between spectra taken from retinal imaging and in vitro reference spectra. This spectral angle, determined by expressing spectra as vectors and computing the angle between them, can be used to capture dissimilarity between two spectral curves. This computation ignores the length of the vectors, which is mainly determined by brightness, and only takes into account spectral features.^43^^,^^78^^,^^79^ The same authors select retinal regions of interest and compare optical densities in different stages of AD in humans.^81^ Alternatively, rather than directly observing differences in spectra, Hadoux et al.^56^ semiautomatically extracted average spectra from regions of interest in the retinal image and feed these as input into a classification model. A variant of linear discriminant analysis produces a “hyperspectral score” that is assumed to be an estimate for retinal amyloid load.^56^ Sharafi et al.^61^ also treat amyloid status as a classification problem and extract a wide range of retinal vessel and texture features from the images, rather than feeding preprocessed spectra directly to the model. These features serve as input to the support vector machine classifier that discriminates amyloid positive patients from amyloid negative individuals.^61^

### Clinical Applications

This section provides an overview of the different ocular phenomena investigated via HSI (Table 2). Next to a variety of studied ophthalmologic conditions, oximetry and AD have gained considerable attention in the HSI field and will therefore be discussed in more depth.

**Table 2.** Retinal HSI Findings as Mentioned in the Literature; Most Cited Ones and Human Studies Are Listed Higher

Ophthalmologic Disease	Pathologic Structures
Primary open angle glaucoma (oximetry)^30^^,^^53^ Dry and wet ARMD^23^ Retinitis pigmentosa^23^ Chronic iridocyclitis^23^ Stargardt's disease^69^ Radiation related retinopathy^59^ Choroidal melanoma^79^ ARMD and ARM^33^^,^^65^^,^^66^^,^^78^	Sub-RPE deposits and peripheral drusen (autofluorescence)^33^^,^^65^ Flecken in Stargardt's disease, drusen in fibulin 3 deficiency and membranoproliferative glomerulonephritis type 2 versus drusen in ARMD^69^ Drusen in ARMD^65^^,^^69^ Optic nerve head drusen^76^ *Drusen in ARMD**^88^*
	**Physiologic Findings**
	Oximetry^26^^,^^36^^,^^50^^,^^51^^,^^57^^,^^58^^,^^60^^,^^76^^,^^77^^,^^87^ Macular pigment (lutein and zeaxanthin)^24^^,^^76^^,^^87^ Cytochrome C in retinal neuron culture^29^ Organoid maturation in stem cells (free NADH, retinol and retinoic acid)^80^ Fluorophore families (as lipofuscin, melanolipofuscin) in RPE^46^^,^^47^ *Oximetry while elevating IOP**^70^*^–^*^73^* *Lymphatic drainage in the eye**^64^* *Oximetry**^25^**^,^**^70^**^,^**^86^*
**Ocular Pathology as a Manifestation of a Systemic Disease**	
(Proliferative) Diabetic retinopathy (oximetry)^32^^,^^62^^,^^77^^,^^83^^,^^89^ Fibulin 3 deficiency^69^ Membranoproliferative glomerulonephritis type 2^69^ Diabetic macular edema^68^ *Ischemic disease (RVO) (oximetry)**^82^*	
**Ocular Findings in a Systemic Disease**	
Hyperbilirubinemia (oximetry)^66^ AD^56^^,^^61^^,^^81^ *Diabetic ONL and reaction to erythropoietin or LCVS1001**^43^**^–^**^45^* *AD and response to γ-GSH**^27^* *Cerebral malaria (oximetry)**^75^*	

The listed phenomena could allegedly be differentiated from healthy individuals or were subject to oximetry alone (indicated). Animal studies are shown in italics.

ARM, age-related maculopathy; LCVS1001 and γ-GSH, compounds not otherwise specified; ONL, outer nuclear layer; RPE, retinal pigment; RVO, retinal vein occlusion.

#### ARMD

HSI of RPE flatmounts in autofluorescence mode revealed recurring spectral patterns, corresponding to lipofuscin and melanolipofuscin.^46^ Similarly, in ARMD flatmounts (RPE and Bruch's membrane), these spectra were found in variable localizations and an emission peak of drusen and sub-RPE deposits was revealed at 510 nm as could be expected (autofluorescence mode, excitation at 436 nm and 480 nm).^33^ Another study successfully quantified the oxidative state of cytochrome C in retinal neurons and therefore proposed HSI as a potential diagnostic marker for retinal degeneration in early ARMD.^29^

#### Diabetes Mellitus

Zamora et al.^68^ found that the retinal spectral variability was larger in patients with diabetes owing to clinically significant macular edema, when compared with healthy individuals. In human nonproliferative diabetic retinopathy, arteriovenous OS difference was found to be lower, but arterial and venular OS were higher compared with controls.^89^ In proliferative diabetic retinopathy, in contrast, arterial OS was lower and venous OS higher than in healthy controls.^83^ A correlation between higher venular OS and several proangiogenic biomarkers in the aqueous humor has been additionally found.^62^

In the spectral range from 636 to 722 nm, the greatest variance was noted in transmittance between normal and diabetic outer nuclear layers in rats.^43^ Retina sections of diabetic rats that were treated with intravitreally injected erythropoietin were found to exhibit intermediate features between normal controls and nontreated diabetic rats.^45^

#### Oximetric Studies (Except for Diabetes Mellitus)

To extract OS levels, saturation is calculated after preprocessing of the hyperspectral cube by (1) taking the ratio between vessel optical density at two wavelengths (one sensitive to saturation, the other isosbestic)^30^^,^^32^^,^^36^^,^^54^^,^^90^; (2) using a formula that combines several features defined to describe the shape of the spectra, decreasing the risk of incidental outliers^70^^–^^73^; or (3) by decomposing optical density into a product including saturation, using the Beert–Lambert law for several wavelengths and solving the resulting set of equations.^23^^,^^25^^,^^26^^,^^28^^,^^63^^,^^82^^,^^86^^,^^87^

Of note, although using hyperspectral hardware and measuring broad spectra, some of these articles solely rely on the two-wavelength method—(1) in previous paragraph—to produce their main findings and one LCTF study that makes use of more than two spectral bands, though with low spectral resolution (20 nm), failed to demonstrate superiority over the older two-wavelength method.^54^^,^^60^^,^^89^

In moderate and severe primary open-angle glaucoma higher mean venular OS was found compared with healthy eyes.^53^ This finding, that the severity of primary open-angle glaucoma is significantly correlated with higher venular OS compared with healthy controls, was confirmed with another set-up.^30^ In healthy participants optic nerve head OS significantly declined during hypoxia,^63^ and total retinal blood flow and arterial OS decreased with increasing arterial partial oxygen pressure.^58^

In healthy cynomolgus monkeys, HSI was able to illustrate that an acute increase of intraocular pressure decreased optic nerve head OS, with partial recovery of OS when a high intraocular pressure was sustained. Based on this ability of the optic nerve head to maintain a certain level of OS despite continued intraocular pressure elevation, the authors suggested a supplemental optic nerve head blood supply through deeper vessels.^70^^,^^73^ This study presented HSI as a noninvasive technique to quantitatively assess the blood OS of both retina and optic nerve head.

OS was determined in patients with retinitis pigmentosa and chronic iridocyclitis.^23^^,^^77^ In patients with radiation-related retinopathy, arteriolar and venular OS were lower in the nondiseased fellow eye compared with the ischemic eye.^59^ In healthy rabbits, except when considering the reperfused state, retinal arteriovenous OS differences were not significant in baseline and ischemic conditions.^25^ While inducing a retinal vein occlusion, arterial and venous OS was found to decrease. These findings were reversible after spontaneous recovery of blood flow.^82^

#### AD

Retinal specimens of APP1/PS1 transgenic mice, a typical animal model for AD, showed significant differences in the spectral region around 500 nm compared with wild-type mice, several months before amyloid plaques became observable in the brain tissue. After treatment with a center specific anti-AD drug candidate, ψ-GSH, retinal (ex vivo) spectra leaned increasingly more toward their saline-treated wild type than their saline-treated transgenic equivalents.^27^ The retinal spectra of APP1/PS1 mice were similar to those observed in postmortem flatmounts of human AD patients, and Rayleigh light scattering at 480 nm, as a measure of amyloid aggregation, reached statistical difference compared with wild-type controls.^67^ Rayleigh scattering is caused by particles up to the size of one tenth of the used wavelength and is inversely proportional to the fourth power of that wavelength.^91^ Because the relatively small, soluble amyloid beta Aβ1–42 aggregates are proven to have an important neurotoxic effect,^92^ 480 nm was used to detect these oligomers because it was the shortest wavelength available in that study. When adapted for in vivo human use, an inverse correlation with the Mini-Mental State Exam score was also seen in those lower wavelengths (430–580 nm).^81^ Hadoux et al. ^56^ successfully trained a classifier to distinguish between amyloid positron emission tomography positive human participants compared with controls based on the hyperspectral signature of the retina. Last, Sharafi et al.^61^ reported a significant hyperspectral difference between retinal tissue (containing vessels) in amyloid positive individuals compared with amyloid negative ones. Elaboration on the data analysis techniques that were used in these studies can be found in the section on Hyperspectral Data Processing. As can be seen from this discussion, many retinal HSI approaches for AD have been suggested, but, notably, none have reached the desired level of technology readiness for clinical trials. The exception perhaps is Hadoux et al.,^56^ who demonstrated a mature HSI technology that has undergone extensive development. Interestingly, no statistically significant differences were found between AD cases and controls on the basis of uncorrected hyperspectral reflectance data, but significant differences were demonstrated when the data were corrected for sources of spectral variability unrelated to AD such as the lens, macular pigment, melanin, and hemoglobin.

#### Miscellaneous Findings

Optic disc drusen were found to be more reflective in the 530- to 580-nm range compared with normal optic disc tissue. Absorption of macular pigment in the 475- to 520-nm range peaked around 490 nm and decreased nearly to zero beyond 500 nm.^76^ In six healthy individuals, an absorbance spectrum with 2 peaks at 460 nm and 490 nm corresponded with the distribution of macular pigment.^24^

Hemoglobin absorption seemed to be greatest at 580 nm, whereas choroidal contrast was best at 650 nm.^28^ In the spectrum with the least difference between arteriovenous optical density ratios (500–580 nm) this ratio can be used as a measure for repeatability.^54^

In one study visible and near-infrared spectrum HSI was able to discriminate choroidal melanoma from other intraocular tumors, based on an increased variation of spectra obtained from melanomas compared with other tumors.^79^

## Discussion

### Barriers and Opportunities for Clinical Implementation

Despite case reports of clinical implementations of HSI, this systematic review highlights the long way ahead before introduction in every day practice. First, there is considerable heterogeneity in HSI setups and most studies are merely proof of concepts. Moreover, some studies suffered from very low sample sizes (Table 1).^33^ Large validation studies are therefore lacking and reproducibility studies could be of value in this respect. As an example, Mordant et al.^52^ failed to assess the reproducibility of their LCTF method owing to a lack of precision and small sample sizes. In contrast, for TLS, sufficient reproducibility was achieved.^57^^,^^58^

Second, consideration should be paid to some inherent limitations of ocular HSI that may hamper its clinical use: Stokes shift, ocular movement, and spatial–spectral resolution issues.

Stokes shift is the spectral shift to lower energy—and thus longer wavelength—between the incident and the emitted light after interaction with an object and depending on the chemical structure of the object. In spectroscopy, this shift refers to the difference between the spectral position of the maximum of the first absorption band and the maximum of the emission. The hyperspectral signature of a retinal sample depends on its molecular composition, which in turn gives rise to Stokes shift. This shift, together with RPE-specific features, is what enables autofluorescence imaging and can therefore be seen as an advantage. However, owing to the large heterogeneity of the retinal tissue, the exact contribution of Stokes shift to the reflectance spectrum cannot yet be predicted, and therefore caution is needed when comparing spectra between studies of different set-up (e.g., LCTF are used both in the illumination^26^^,^^28^^,^^49^ as well as the imaging path).

Like other scanning techniques (Fig. 1B1), the time–sequential methods (nonsnapshot) are subject to ocular drift, microsaccades, and tremor. For this reason, software for coregistration of retinal landmarks and automated realignment of subsequent images is indispensable to achieve a higher signal-to-noise ratio.^48^ However, as a result of coregistration and realignment, resolution of up to 4 nm can be reached for Fourier based scanning techniques and up to 2 nm for the most commonly used techniques such as LCTF, TLS, and PGP.^27^^,^^43^^,^^44^^,^^48^^,^^68^^,^^79^ The higher resolution reached with the latter techniques comes at the cost of other drawbacks, which have to be considered when selecting the most appropriate HSI technique: LCTF are said to be prone to low optical throughput, and polarization sensitivity,^26^^,^^49^^,^^54^ whereas image artefacts caused by the reflectivity in the mirror facets are noted in dispersion based methods.^76^ Additionally PGP are substantially large, making them unsuitable for handheld applications^86^; however, their short processing time, independence of unfocused background light, and low cost are, in contrast, advantageous.^23^ Importantly, the issues related to (ocular) movements can alternatively be overcome by CTIS, fiber optics, IMS, and SRDA, being snapshot methods with acquisition times of less than 50 ms.^23^^–^^25^^,^^36^^,^^50^^,^^76^^,^^82^^,^^83^^,^^85^^–^^87^

Finally, besides the Stokes shift and ocular movements, a third issue hampering translation of HSI to clinical practice, is the spatial–spectral resolution and acquisition time trade-off. Indeed, at least one of these three is often compromised: the hyperspectral cube of techniques that consume more time to image the retina are characterized by a higher resolution (Table 1 and Fig. 2). This brings us to the question whether snapshot techniques like CTIS and SRDA possess enough resolution to have a clinical impact. In comparison, CTIS achieved higher spectral resolution than SRDA and successfully explored the spatial distribution of macular pigments in vivo, but came at the cost of high computational complexity and calibration difficulty. In contrast, SRDA was used for OS measurement with good reproducibility.^87^ The limited spectral resolution of SRDA could hypothetically be resolved via augmenting the number of fabricated layers of Fabry–Perrot cavity filters on complementary metal oxide semiconductor pixels, inevitably decreasing spatial resolution, or alternatively with multiple dielectric layer cavities, possibly also extending the wavelength range. More studies are due to determine whether upgrading these complementary metal oxide semiconductor chips will overcome the spatial–spectral resolution and acquisition time trade-off.^86^^,^^87^

### Developments and Future Applications

Despites these hurdles still standing in between bench and bedside, the last decade of HSI research and development, and especially the recent successes with HSI for Alzheimer's diagnosis and oximetry, shows the potential of HSI for ophthalmologic practice. Overcoming the aforementioned challenges would propel the use of HSI applications in the clinic and may also foster the development of novel HSI tools. One such development is HSI as an alternative for fluorescein angiography.^77^ Indeed, a number of studies on dispersive arrays and TLS extended their spectrum in the near-infrared field.^27^^,^^49^^,^^56^^,^^61^^,^^70^^,^^78^^,^^79^^,^^81^ Near-infrared light does not damage ocular tissue and is strongly absorbed by water (subretinal fluid) and more important, melanin, although it is not affected by the ocular transparent media and macular pigment. Therefore, it is supposed to be an ideal band to examine choroidal neovascularization, even in the presence of cataract.^78^ As such, it may be an alternative for fluorescein angiography.

The focus on HSI thus far, and the reported proofs of concept, has been dedicated to the acquisition of a single hyperspectral cube of the retina. As a next step, higher frame rates and independence from long processing times (e.g., in CTIS) could make SRDA and IMS candidates for instantaneous and continuous visualization.^86^ This feature enables real-time information and could be of particular interest during surgery; for example, real-time HSI is already described for skin, kidney, and brain surgery.^93^ For this application, the right spatial dimension has to be considered: dispersive arrays are less convenient for handheld use, similar to the bulky CTIS methods, when compared with the compactness of SRDA.^23^^–^^25^^,^^36^^,^^76^^,^^82^^,^^83^^,^^86^^,^^87^

Finally, high-resolution and adaptive imagery settings are needed for intracellular visualization, cultures, histology, and, thus, experimental research. Combined with microscopes, TLS and LCTF might be more suited for dynamic cultures, whereas scanning dispersive arrays are more appropriate for static specimens as moving organelles could intuitively cause errors.^43^^,^^45^^,^^64^

## Conclusions

HSI in ophthalmology remains in its infancy. Although many studies have shown its potential, the fields still suffers from variability in hardware and software, lack of comparison thereof, and lack of sufficiently large sample sizes. Larger and structured experiments are needed to explore the true potential of HSI in ophthalmology practice, narrowing down on the number of technical setups. At present, the research field is very scattered. Most HSI is being performed in the field of retinal oximetry yet recent studies have also shown promising results for HSI in AD diagnosis, disease monitoring and research. Hyperspectral retinal imaging might be worth the wave, but do not expect an easy glide.

## Supplementary Material

Supplement 1

Supplement 2
