# Supplementary material for: Hyperspectral Imaging and the Retina: Worth the Wave?
Source: Transl Vis Sci Technol. 2020 Aug 5;9(9):9. doi: 10.1167/tvst.9.9.9 (PMC7442879; doi:10.1167/tvst.9.9.9)
Supplement: Supplement 1 [file tvst-9-9-9_s001.pdf]

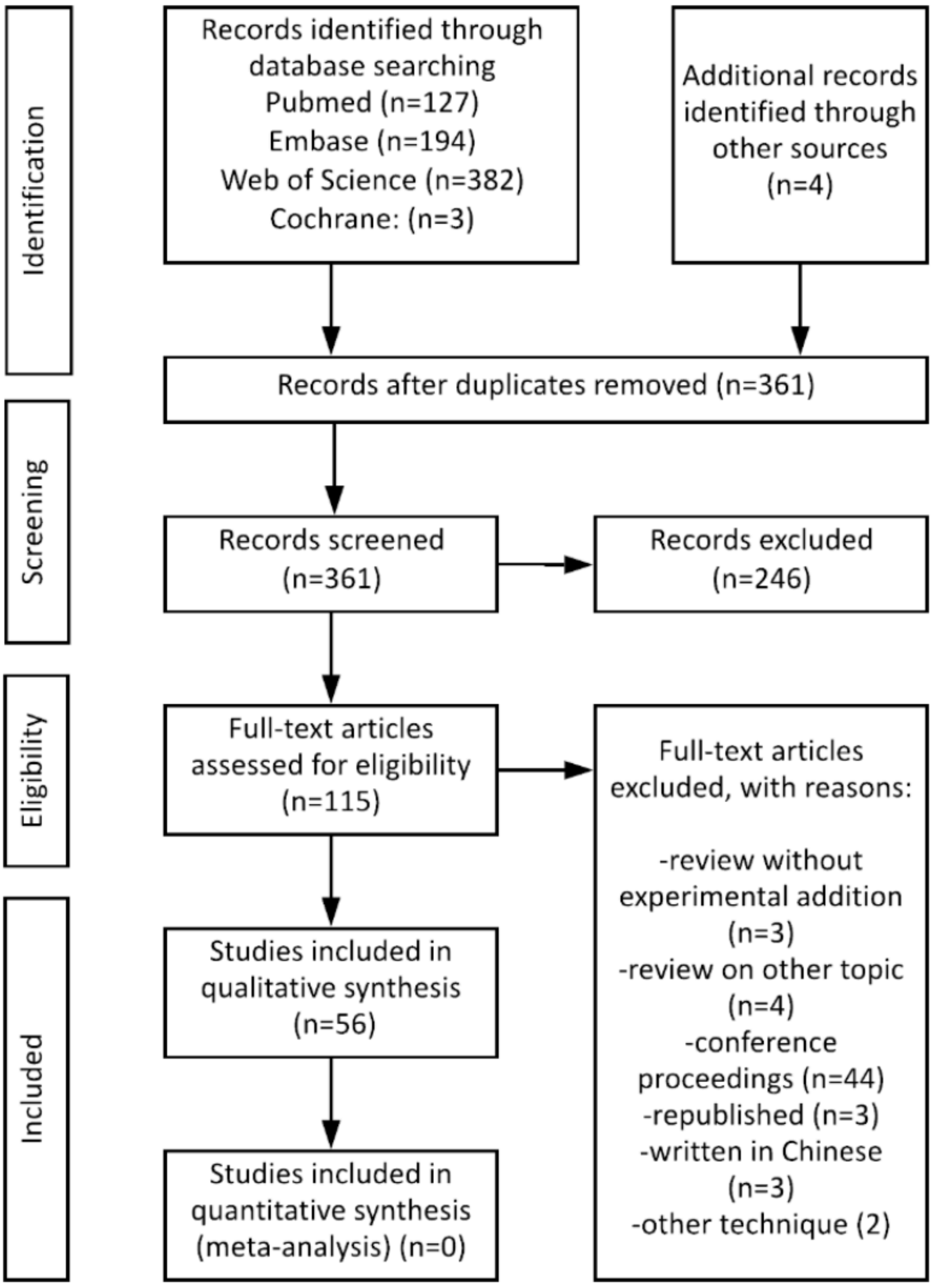

Fig. S1. Literature search: PRISMA Consort flow diagram.

n: number of articles. According to The PRISMA Statement 2009.[38]
