# Supplementary material for: Hyperspectral Imaging and the Retina: Worth the Wave?
Source: Transl Vis Sci Technol. 2020 Aug 5;9(9):9. doi: 10.1167/tvst.9.9.9 (PMC7442879; doi:10.1167/tvst.9.9.9)
Supplement: Supplement 2 [file tvst-9-9-9_s002.docx]

# Supplementary material: Search strategy, included publications, bias and quality assessment

## S1 Boolean operators

### S1.1 PubMed (Medline)

((“eye”[MeSH] OR eye*[tiab] OR “ophthalmology”[MeSH] OR ophthalmology*[tiab] OR "retinal vessels"[MeSH] OR retina*[tiab] OR fundus*[tiab] OR macula*[tiab] OR fovea*[tiab])) AND hyperspectral*[tiab]

### S1.2 Embase

Concept 1: 'eye'/exp OR 'eye*’:ti,ab OR 'eye disease'/exp OR 'ophthalmology'/exp OR 'ophthalmolog*':ti,ab OR 'retina*':ti,ab OR 'retina disease'/exp OR 'retina blood vessel'/exp OR 'retinal vascular disease'/exp OR 'eye fundus'/exp OR 'fundus*':ti,ab OR 'retina maculopathy'/exp OR 'maculopath*':ti,ab OR 'retina fovea'/exp OR 'fovea*':ti,ab OR ‘macula*’:ti,ab

Concept 2: 'hyperspectral imaging'/exp OR 'hyperspectral imaging system'/exp OR ‘hyperspectral*’:ti,ab

Combine

### S1.3 Web of Science

Concept 1: eye* OR ophthalmolog* OR retina* OR fundus* OR macula* OR maculopath* OR fovea*

Concept 2: hyperspectral*

Combine

### S1.4 Cochrane Database of Systematic Reviews

Concept 1: hyperspectral

Concept 2: retina

Combine

*S2 Included papers*

**Table S1.** Included papers. All included articles listed in alphabetical order.

| **Author** | **Year** | **Title** | **Magazine** |
| --- | --- | --- | --- |
| **Alabboud et al.^51^** | 2007 | New spectral imaging techniques for blood oximetry in the retina | Proceedings of SPIE |
| **Beach et al.^71^** | 2007 | Oxygen saturation in optic nerve head structures by hyperspectral image analysis | Current Eye Research |
| **Beach et al.^72^** | 2009 | A simple model of oxygen diffusion out of the retinal artery | Proceedings of SPIE |
| **Ben Ami et al.^46^** | 2016 | Spatial and spectral characterization of human retinal pigment epithelium fluorophore families by ex vivo hyperspectral autofluorescence imaging | Translational Vision Science and Technology |
| **Browne et al.^80^** | 2017 | Structural and functional characterization of human stem-cell-derived retinal organoids by live imaging | Investigative Ophthalmology & Visual Science |
| **Davis et al.^69^** | 2007 | Identification of spectral phenotypes in age-related macular degeneration patients | Proceedings of SPIE |
| **Desjardins et al.^63^** | 2016 | Preliminary investigation of multispectral retinal tissue oximetry mapping using a hyperspectral retinal camera | Experimental Eye Research |
| **Dey et al.^65^** | 2019 | Tensor decomposition of hyperspectral images to study autofluorescence in age-related macular degeneration | Medical Image Analysis |
| **Dwight et al.^23^** | 2016 | Hyperspectral image mapping spectrometry for retinal oximetry measurements in four diseased eyes | International Ophthalmology Clinics |
| **Dwight et al.^77^** | 2019 | A dye-free analog to retinal angiography using hyperspectral unmixing to retrieve oxyhemoglobin abundance | Translational Vision Science & Technology |
| **Fawzi et al.^24^** | 2011 | Recovery of macular pigment spectrum in vivo using hyperspectral image analysis | Journal of Biomedical Optics |
| **Francis et al.^66^** | 2011 | Hyperspectral retinal imaging with a spectrally tunable light source | Proceedings of SPIE |
| **Gao et al.^76^** | 2012 | Snapshot hyperspectral retinal camera with the image mapping spectrometer (IMS) | Biomedical Optics Express |
| **Harvey et al.^49^** | 2002 | Hyperspectral imaging for the detection of retinal diseases | Proceedings of SPIE |
| **Hadoux et al.^56^** | 2019 | Non-invasive in vivo hyperspectral imaging of the retina for potential biomarker use in Alzheimer’s disease | Nature Communications |
| **Hirohara et al.^54^** | 2007 | Validity of retinal oxygen saturation analysis: hyperspectral imaging in visible wavelength with fundus camera and liquid crystal wavelength tunable filter | Optical Review |
| **Jaime et al.^82^** | 2012 | Acute variations in retinal vascular oxygen content in a rabbit model of retinal venous occlusion | Plos One |
| **Johnson et al.^36^** | 2007 | Snapshot hyperspectral imaging in ophthalmology | Journal of Biomedical Optics |
| **Kaluzny et al.^87^** | 2017 | Bayer filter snapshot hyperspectral fundus camera for human retinal imaging | Current Eye Research |
| **Kameyama et al.^79^** | 2015 | Noninvasive diagnostics supporting system for choroidal melanoma: a pilot study | Japanese Journal of Ophthalmology |
| **Kashani et al.^25^** | 2011 | Hyperspectral computed tomographic imaging spectroscopy of vascular oxygen gradients in the rabbit retina in vivo | Plos One |
| **Kashani et al.^83^** | 2014 | Noninvasive assessment of retinal vascular oxygen content among normal and diabetic human subjects: a study using hyperspectral computed tomographic imaging spectroscopy | Retina |
| **Khoobehi et al.^70^** | 2004 | Non-invasive measurement of oxygen saturation in optic nerve head tissue | Optical Diagnostics and Sensing IV |
| **Khoobehi et al.^73^** | 2009 | Oxygen saturation changes in the optic nerve head during acute intraocular pressure elevation in monkeys | Proceedings of SPIE |
| **Khoobehi et al.^74^** | 2011 | Enhanced oxygen saturation in optic nerve head of non-human primate eyes following the intravitreal injection of NCX 434, an innovative nitric oxide-donating glucocorticoid | Journal of Ocular Pharmacology and Therapeutics |
| **Khoobehi et al.^50^** | 2012 | Determination of oxygen saturation of the optic nerve head and overlying artery and vein using a snapshot multi-spectral imaging system | Proceedings of SPIE |
| **Khoobehi et al.^84^** | 2012 | Snapshot hyperspectral imaging to measure oxygen saturation in the retina using fiber bundle and multi-slit spectrometer | Proceedings of SPIE |
| **Khoobehi et al.^85^** | 2014 | A new snapshot hyperspectral imaging system to image optic nerve head tissue | Acta Ophthalmologica |
| **Li et al.^43^** | 2007 | New microscopic pushbroom hyperspectral imaging system for application in diabetic retinopathy research | Journal of Biomedical Optics |
| **Li et al.^44^** | 2008 | Microscopic hyperspectral imaging studies of normal and diabetic retina of rats | Science in China Series C: Life Sciences |
| **Li et al.^45^** | 2010 | Quantitative analysis of protective effect of erythropoietin on diabetic retinal cells using molecular hyperspectral imaging technology | IEEE Transactions on Biomedical Engineering |
| **Li et al.^86^** | 2017 | Snapshot hyperspectral retinal imaging using compact spectral resolving detector array | Journal of Biophotonics |
| **Liu et al.^75^** | 2012 | Spectral reflectance of the ocular fundus as a diagnostic marker for cerebral malaria | Proceedings of SPIE |
| **Mordant et al.^52^** | 2011 | Validation of human whole blood oximetry, using a hyperspectral fundus camera with a model eye | Investigative Ophthalmology & Visual Science |
| **Mordant et al.^26^** | 2011 | Spectral imaging of the retina | Eye |
| **Mordant et al.^53^** | 2014 | Oxygen saturation measurements of the retinal vasculature in treated asymmetrical primary open-angle glaucoma using hyperspectral imaging | Eye |
| **More et al.^27^** | 2015 | Hyperspectral imaging signatures detect amyloidopathy in Alzheimer’s mouse retina well before onset of cognitive decline | ACS Chemical Neuroscience |
| **More et al.^67^** | 2016 | Early detection of amyloidopathy in Alzheimer's mice by hyperspectral endoscopy | Investigative Ophthalmology & Visual Science |
| **More et al.^81^** | 2019 | In Vivo Assessment of Retinal Biomarkers by Hyperspectral Imaging: Early Detection of Alzheimer’s Disease | ACS Chemical Neuroscience |
| **Nourrit et al.^28^** | 2010 | High-resolution hyperspectral imaging of the retina with a modified fundus camera | Journal français d'Ophtalmologie |
| **Patel et al.^57^** | 2013 | A prototype hyperspectral system with a tunable laser source for retinal vessel imaging | Investigative Ophthalmology & Visual Science |
| **Rose et al.^58^** | 2016 | Intervisit repeatability of retinal blood oximetry and total retinal blood flow under varying systemic blood gas oxygen saturations | Investigative Ophthalmology & Visual Science |
| **Rose et al.^59^** | 2018 | Retinal perfusion changes in radiation retinopathy | Acta Ophthalmologica |
| **Schweizer et al.^29^** | 2012 | Hyperspectral imaging - a new modality for eye diagnostics | Biomedizinische Technik/ Biomedical Engineering |
| **Shahidi et al.^60^** | 2013 | Regional variation in human retinal vessel oxygen saturation | Experimental Eye Research |
| **Shahidi et al.^30^** | 2017 | Retinal oxygen saturation in patients with primary open-angle glaucoma using a non-flash hyperspectral camera | Current Eye Research |
| **Sharafi et al.^61^** | 2019 | Vascular retinal biomarkers improves the detection of the likely cerebral amyloid status from hyperspectral retinal images | Alzheimer’s & Dementia: Translational Research & Clinical Interventions |
| **Smith et al.^47^** | 2014 | Simultaneous decomposition of multiple hyperspectral data sets: signal recovery of unknown fluorophores in the retinal pigment epithelium | Biomedical Optics Express |
| **Tam et al.^64^** | 2011 | Quantum dots trace lymphatic drainage from the mouse eye | Nanotechnology |
| **Tayyari et al.^32^** | 2015 | Retinal blood flow and retinal blood oxygen saturation in mild to moderate diabetic retinopathy | Investigative Ophthalmology & Visual Science |
| **Tayyari et al.^62^** | 2019 | Retinal blood oxygen saturation and aqueous humour biomarkers in early diabetic retinopathy | Acta Ophthalmologica |
| **Tong et al.^33^** | 2016 | Hyperspectral autofluorescence imaging of drusen and retinal pigment epithelium in donor eyes with age-related macular degeneration | Retina |
| **Truitt et al.^48^** | 2000 | Hyperspectral fundus imager | Proceedings of SPIE |
| **Wang et al.^88^** | 2019 | Drusen diagnosis comparison between hyperspectral and color retinal images | Biomedical Optics Express |
| **Yamauchi et al.^78^** | 2012 | Novel automated screening of age-related macular degeneration | Japanese Journal of Ophthalmology |
| **Zamora et al.^68^** | 2004 | Hyperspectral imaging analysis for ophthalmic applications | Proceedings of SPIE |

## S3 Bias assessment

The NOS was performed on eight included case control studies (Table S2) in order to quantify their overall risk of bias. Nearly all studies included a relatively low number of study subjects and overall NOS scores ranged from 4 up to 8 (out of 10).

QUADAS-2 was executed on four included studies that primarily examined the diagnostic accuracy of hyperspectral imaging (Table S3). Kameyama and colleagues focused on the diagnosis of intraocular tumors,^79^ whereas the other three investigated the relationship between hyperspectral retinal imaging and Alzheimer’s disease (AD).^56,61,81^ Studies tend to be at risk of bias, mainly based on index test, flow and timing criteria.

**Table S2.** NOS assessment.

| NOS | Selection | | | | Comparability | Exposure | | | Stars /10 |
| --- | --- | --- | --- | --- | --- | --- | --- | --- | --- |
| Study | **Case definition** | **Representativeness** | **Controls' selection** | **Controls' definition** | **Controls for** | **Ascertainment** | **Same method** | **Non-response** | **.** |
| *Example* | *independent validation* | *consecutive series* | *community* | *no history of disease* | *2 factors* | *secure record + blinded* | *yes* | *same rate* | *10* |
| Shahidi et al.^30^ | **✓** | not stated | no description | no description | not stated | **✓** secure record | **✓** | **✓** | **4** |
| Tayyari et al.^32^ | **✓** | not stated | no description | **✓** | **✓** age | medical report only | **✓** | **✓** | **6** |
| Rose et al.^59^ | **✓** | **✓** | hospital | **✓** | **✓** same person | **✓** secure record | **✓** | **✓** | **8** |
| Zamora et al.^68^ | **✓** | not stated | no description | **✓** | **✓** age | medical report only | **✓** | **✓** | **5** |
| Kashani et al.^83^ | **✓** based on record or self-report | not stated | hospital | **✓** | **✓** no significant differences | medical report only | **✓** | **✓** | **6** |
| Davis et al.^69^ | **✓** | not stated | hospital | no description | **✓** age | medical report only | **✓** | **✓** | **4** |
| Mordant et al.^53^ | **✓** | not stated | hospital | **✓** | not stated | medical report only | **✓** | **✓** | **5** |
| Yamauchi et al.^78^ | **✓** | not stated | no description | **✓** | not stated | medical report only | **✓** | **✓** | **5** |

According to the Newcastle-Ottawa Quality Assessment Scale for case-control studies or cohort studies.^38^ A checkmark is only placed whenever the requirement is met. For requirement 5 (and 6), a detail is written of each factor for which is controlled. Whenever two eyes of the same person were compared, the study was rated 2 stars for this topic

**Table S3.** QUADAS-2 assessment.

| QUADAS-2 | RISK OF BIAS | | | | APPLICABILITY CONCERNS | | |
| --- | --- | --- | --- | --- | --- | --- | --- |
| Study | **Patient selection** | **Index test** | **Reference standard** | **Flow and timing** | **Patient selection** | **Index test** | **Reference standard** |
| Kameyama et al.^79^ |  |  |  |  |  |  |  |
| Hadoux et al.^56^ |  |  |  |  |  |  |  |
| More et al.^81^ |  |  |  |  |  |  |  |
| Sharafi et al.^61^ |  |  |  |  |  |  |  |

According to the Quality Assessment of Diagnostic Accuracy Studies-2 (QUADAS-2) tool.^38^

|  | Low risk |  | High risk |  | Unclear |
| --- | --- | --- | --- | --- | --- |

Color code:
